# Reply: Concussions Are Associated With Increases in Blood Pressure and Cardiovascular Risk in American-Style Football Athletes

**DOI:** 10.1016/j.jacadv.2025.101950

**Published:** 2025-07-03

**Authors:** Austin J. Rim, Jonathan H. Kim

**Affiliations:** Emory Clinical Cardiovascular Research Institute, Emory University School of Medicine, Atlanta, Georgia, USA

We appreciate the thoughtful insights and positive comments from Dr Skalidis and colleagues regarding our recent study on the association between concussions and early cardiovascular risk present among collegiate American-style football (ASF) players.^1^ Their comments highlight the potential overarching impact of our findings, specifically the need to consider sport-associated concussion as a novel cardiovascular risk factor. We concur with these authors that the present study sets the stage for several promising avenues for future research, which could improve the long-term neurologic, neurocognitive, and cardiovascular health among ASF athletes who sustain concussion.

In response to the additional questions posed by the authors, we believe our findings suggest that more intensive blood pressure monitoring may be warranted as part of routine postconcussive clinical management among competitive athletes who sustain sport-related concussion. Routine resting blood pressure monitoring is noninvasive and easily conducted within an athletic training room. Although current guidelines suggest that isolated orthostatic vital signs measured during the subacute phase postconcussion (3-30 days postinjury)^2^ may be warranted, there are currently no recommendations on the need to assess resting blood pressure for any length of time after a sustained sport-associated concussion. In general, an improved focus on blood pressure surveillance should be emphasized among all elite ASF athletes, and these current data suggest that sustained concussions may be another risk factor, in addition to weight gain, sleep apnea, and nonsteroidal anti-inflammatory overuse, all uniquely prevalent in this population. It remains uncertain the appropriate duration of time blood pressure surveillance would be necessary after sport-related concussion and if additional risk stratification with other clinical tests, beyond blood pressure surveillance, would be necessary in order to affect health outcomes. These uncertainties warrant future study.

Ultimately, future prospective studies inclusive of larger cohorts of ASF athletes and athletes represented by other sporting disciplines are required to affirm our findings. In addition, the long-term impact of concussion on cardiovascular phenotypes, and the effects of longitudinal postconcussion blood pressure surveillance on outcomes remain uncertain. Perhaps even more importantly, whether interventions to reduce blood pressure during the recovery phase after sport-associated concussion improve outcomes is a critical unanswered question. We completely agree with Skalidis et al on the importance of all of these issues as we strive to understand the implications of sport-associated concussion on numerous health outcomes, including cardiovascular health.

## References

[1] Rim A.J., Liu C., Jackson M. (2025). Concussions are associated with increases in blood pressure and cardiovascular risk in American-style football athletes. JACC Adv.

[2] Patricios J.S., Schneider K.J., Dvorak J. (2023). Consensus statement on concussion in sport: the 6th International Conference on Concussion in Sport-Amsterdam, October 2022. Br J Sports Med.

